# All Patients With Common Variable Immunodeficiency Disorders (CVID) Should Be Routinely Offered Diagnostic Genetic Testing

**DOI:** 10.3389/fimmu.2019.02678

**Published:** 2019-11-22

**Authors:** Rohan Ameratunga, Klaus Lehnert, See-Tarn Woon

**Affiliations:** ^1^Department of Virology and Immunology, Auckland City Hospital, Auckland, New Zealand; ^2^Department of Clinical Immunology, Auckland City Hospital, Auckland, New Zealand; ^3^Department of Molecular Medicine and Pathology, Faculty of Medical and Health Sciences, University of Auckland, Auckland, New Zealand; ^4^School of Biological Sciences, University of Auckland, Auckland, New Zealand

**Keywords:** CVID - common variable immunodeficiency disorders, hypogammaglobinaemia, genetic sequence analysis, transient hypogammaglobulinemia of adulthood (THA), transient hypogammaglobulinemia of infancy

## Introduction

Common Variable Immunodeficiency Disorders (CVID) are a rare group of primary immunodeficiency disorders (PIDs) where late onset antibody failure leads to immune system failure (1). Onset of symptoms can occur from early childhood to the eighth decade or later (2). Current estimates suggest a prevalence between 1: 25 000 to 1: 100 000 in Caucasians (3, 4). For reasons that are unclear, CVID appears to be less frequent in Asian and African populations, although there may be ascertainment bias.

The majority of patients with CVID present with recurrent and severe infections. Untreated, patients are predisposed to chronic suppuration of the respiratory tract, often resulting in chronic sinus disease and bronchiectasis. Approximately 25% of CVID patients suffer autoimmune or inflammatory sequelae, consequent to immune dysregulation (5). There is also an increased risk of malignancy (6).

There is no single clinical feature or laboratory test, which is pathognomonic for CVID. The identification of CVID therefore relies on diagnostic criteria. There are currently three new sets of diagnostic criteria for CVID (7–9). The Ameratunga et al. (7) criteria require symptomatic primary hypogammaglobulinemia with relevant laboratory tests to establish the diagnosis. The threshold for IgG was set at 5 g/l for adults. The revised European Society for immunodeficiencies (8) are similar to the Ameratunga et al. criteria.

The most recent International Consensus Document (9) CVID criteria claim to be able to make a definite diagnosis on the basis of a single abnormal vaccine challenge result in a patient with primary hypogammaglobulinemia (9). The latter two criteria have set the threshold for IgG at 2 sd below the mean. Although immunoglobulin levels do not follow a Gaussian distribution (10), this is generally accepted as an IgG below 7 g/l. These criteria also exclude late onset combined immunodeficiency (LOCID) from the diagnosis. The immunoglobulin levels are also required to be repeated in these latter criteria.

There is ongoing debate about the utility these diagnostic criteria, the variability of IgG levels over time, the unreliability of vaccine challenge responses and flow cytometry in the diagnosis of CVID. As discussed below, this variability in protein-based assays is a strong argument for genetic testing of all patients with a CVID phenotype.

## Genetics of CVID and CVID-Like Disorders

By definition the causes of CVID are unknown. In 2003, the first genetic defect was identified in Germany (11). Mutations of the Inducible T cell co-stimulator (*ICOS*), which plays a critical role in T and B cell communication, were discovered in patients with a CVID phenotype (11). There was a founder effect as all affected individuals in the Black Forest area shared the identical mutation. Different mutations of *ICOS* were subsequently identified in other parts of the world, confirming allelic heterogeneity (12).

Two years later, mutations of the T cell activator, calcium modulator and cyclophilin ligand interactor (*TNFRSF13B/* TACI) were discovered (13). This molecule plays an important role in B cell signaling and immunoglobulin isotype switching. Mutations of *TNFRSF13B/*TACI were initially thought to cause CVID but subsequently it became apparent that identical mutations were also found in the general population at a frequency far greater than the incidence of CVID. It is now thought that mutations of *TNFRSF13B/*TACI predispose to CVID or have a disease modifying effect on the disorder (14).

We and others have suggested mutations associated with CVID should be categorized according to whether they cause the disorder or whether they modify or predispose to the condition (7, 15). Apart from *TNFRSF13B*/TACI, mutations of other genes including BAFFR (*TNFRSF13C*), TWEAK (*TNFSF12*), *MSH5*, and TRAIL (*TNFSF10*) are also thought to predispose to, or modify the disease severity of patients with CVID.

In contrast, mutations of genes such as *NFKB1, NFKB2, CTLA-4, TCF3* etc. are more likely to cause the condition (16, 17). Such patients are removed from the broad category of CVID and are deemed to have a PID caused by a specific mutation. We have suggested conditions with causative mutations should be termed “CVID-like” disorders, given the close phenotypic overlap with CVID (18). None of the current diagnostic criteria for CVID allow the diagnosis if a known disorder is identified (7–9, 18). This is the basis for excluding patients with a causative mutation from the umbrella diagnosis of CVID. Since the discovery of *ICOS* mutations, ~30 genetic defects have been shown to modify disease severity, predispose to CVID or alternatively cause CVID-like disorders (19–21).

CVID is genetically complex. Locus heterogeneity (genocopy) is a major feature of CVID-like disorders, making it difficult to identify the affected gene purely on clinical grounds. Mutations of several genes can result in the classical phenotype of late onset antibody failure leading to recurrent and severe infections as well as autoimmunity (19).

Although clinical identification of individual CVID-like disorders is difficult, there may be subtle clues such as the presence of alopecia in combination with pituitary dysfunction, which are indicative of *NFKB2* defects (19). In other cases, a careful history may reveal severe autoimmunity, which may suggest *PIK3CD* mutations, causing activated protein kinase 3D syndrome (APDS) or *CTLA-4*/*LRBA* mutations (22). The presence of vasculitis in the context of hypogammaglobulinemia might indicate *ADA2* deficiency (19). In most cases however, such clues are absent.

Similarly, phenotypic heterogeneity makes diagnosis difficult as the clinical manifestations can vary widely, even within the same family carrying the identical mutation. We have recently described the pleomorphic clinical presentation of a family with *NFKB1* deficiency (23). One heterozygous brother carrying the mutation was asymptomatic with normal immunoglobulins, while his heterozygous sister had severe disease with features of late onset combined immunodeficiency (LOCID) (23). We have used our CVID disease severity score (CDSS) to quantify the phenotypic severity of individual family members (24). The phenotypic heterogeneity may be the result of variable penetrance and expressivity, epigenetic influences or epistasis caused by gene-gene interactions.

As noted in the case of *ICOS* deficiency, CVID-like disorders also manifest allelic heterogeneity where different mutations of the same gene can result in a similar phenotype. Because of genetic and phenotypic heterogeneity, there has been understandable reluctance to routinely sequence CVID patients because of the low yield (25). Serial Sanger sequencing of an ever-increasing list of individual genes was not an efficient use of valuable resources (25).

Given the rapid progress in the understanding of these conditions in recent years, we believe there is now a strong case for routine diagnostic genetic testing of patients with a CVID phenotype (Table 1). This change in approach is both the result of identifying increasing numbers of genetic defects as well as advances in technology, particularly NGS. We have previously discussed diagnosing CVID in the era of genome sequencing (19). In this current viewpoint article, we have incorporated new information, mostly from our recent studies, to strengthen the arguments for routine diagnostic sequencing of patients with a CVID phenotype (26, 27). This article will serve as the evidence base for what is becoming routine practice in the care of CVID patients. It will assist clinical services in implementing such a strategy.

**Table 1:** The utility of genetic testing for patients with a CVID phenotype.

**Establishing the diagnosis**
Confirming the clinical diagnosis of a CVID-like disorder
Identifying novel presentations of other CVID-like disorders eg as LOCID
Identifying atypical presentations of other PIDs with hypogammaglobulinemia eg XLP
Distinguishing genetic from acquired disorders eg drug-induced hypogammaglobulinemia Identifying digenic disorders THA-Variability of IgG levels over time: some of these patients may have CVID-like disorders Differences in diagnostic criteria for CVID: the presence of a CVID-like disorder will obviate the need to apply CVID diagnostic criteria. Identifying CVID-like disorders in patients who have already developed malignancy Identifying CVID-like disorders in patients on SCIG/IVIG or immunosuppression
**Treatment**
Offering early SCIG/IVIG treatment for individuals carrying causative mutations Identifying specific treatment options eg abatacept for *CTLA-4/LRBA* deficiency
Identifying patients who may benefit from gene based therapy in the future
**Prognosis**
Asymptomatic patients with monogenic defects have a high probability of symptomatic disease, leading to long-term SCIG/IVIG treatment May distinguish patients with THI, who may not recover till adulthood where some have impaired vaccine responses
**Pre-symptomatic testing**
Where presymptomatic diagnosis (at any age) is not possible with protein based tests eg patients with CVID-like disorders who are asymptomatic with normal immunoglobulins
Diagnosis in infancy where conventional diagnostic tests are unreliable eg because of transplacentally acquired IgG levels
**Screening**
Cascade screening of at-risk relatives with or without symptoms after genetic counseling Identifying mutations from tissue samples from deceased relatives Identifying mutations from Guthrie cards from deceased relatives
**PID prevention**
Prenatal diagnosis with chorionic villus sampling (CVS)
Pre-implantation genetic diagnosis (PGD)
**Research**
Characterizing the role of molecules in cellular function
Assisting with the classification of primary immunodeficiency disorders
Identification of new genetic defects with trio analysis Investigating animal models of CVID-like disorders Identifying epistasis caused by digenic (or oligogenic) disorders

*Most of the clinical scenarios are described in the text. LOCID - late onset combined immunodeficiency, SCIG/IVIG - subcutaneous of intravenous immunoglobulin treatment, THA - transient hypogammaglobulinemia of adulthood, THI - transient hypogammaglobulinemia of infancy, XLP - X-linked lymphoproliferative disorder*.

## NGS Allows Efficient Sequencing of Genes from Disorders With Locus Heterogeneity

Over the last decade, NGS has revolutionized the approach to molecular diagnosis. Multiple genes can now be sequenced simultaneously, with either gene panels or by Whole Exome Sequencing (WES) and targeted analysis (19). In non-consanguineous populations, the causative mutation may be identified in ~25% of CVID patients (28). The diagnostic yield is much higher in kindreds with more than one affected individual, founder populations or those with high rates of consanguinity (29–31). In some cohorts, the presence of parental consanguinity was associated with more severe disease (32).

The advent of NGS is the principal reason for the feasibility of routine genetic sequencing of patients with CVID-like disorders, who have locus heterogeneity (33). A similar approach is now being undertaken for other disorders with locus heterogeneity such at atypical hemolytic uremic syndrome (aHUS) and hemophagocytic lymphohistiocytosis (HLH) (34).

Although there was initial concern about copy number variants contributing to CVID (35) a more recent paper did not support these initial observations (36). Therefore WES is a reasonable option for investigating these patients.

We next discuss specific advantages as well as potential disadvantages undertaking routine diagnostic sequencing of patients with a CVID phenotype.

## Specific Advantages of Identifying the Causative Mutation in Patients with CVID-Like Disorders (Table 1)

Identifying the causative genetic defect is now the standard of care of PID patients (25). We have outlined the many overlapping advantages (and some disadvantages) in identifying the underlying genetic defect in PIDs (37–39). We have listed in detail the specific advantages of genetic diagnosis in CVID-like disorders in Table 1.

Identification of a causative mutation will confirm the presence of a CVID-like disorder and will enable diagnosis of patients with atypical presentations. This is particularly important given the genetic and phenotypic heterogeneity in CVID-like disorders, outlined above.

Some patients with well-characterized PIDs such as X-linked lymphoproliferative (XLP) disorder or *STAT3* mutations can present rarely with predominant hypogammaglobulinemia. If other characteristic features of these disorders are not obvious, such atypical presentations may cause confusion with CVID. Given there may be specific treatments for these conditions, early identification is of paramount importance. Pre-emptive bone marrow transplantation prior to EBV infection in pre-symptomatic male relatives, carrying the mutation, can be life-saving in XLP (40). NGS will rapidly identify the majority of “non-CVID” patients presenting with hypogammaglobulinemia. We distinguish PIDs such as XLP and *STAT3* mutations, which do not typically present with antibody deficiency (41–43) from disorders such as *NFKB1* mutations which, most often present with hypogammaglobulinemia, that are more appropriately termed CVID-like disorders (19).

Identification of the mutation will offer prognostic information. We have recently shown that many children with transient hypogammaglobulinemia of infancy (THI) do not recover until early adulthood (27). CVID/CVID-like disorders are thus the principal differential diagnosis until patients with THI recover. Identification of a causative mutation in a child with persistent hypogammaglobulinemia will exclude THI and will indicate the patient is likely to require long-term subcutaneous or intravenous immunoglobulin (SCIG/IVIG) therapy. As noted above, genetic sequencing of children with severe symptomatic immunodeficiencies is now the standard of care.

Hypogammaglobulinemia can be caused by a wide range of non-immunological disorders and it can sometimes be difficult to exclude these secondary causes. If a causative genetic defect is identified, this will exclude secondary causes, such as anticonvulsant drugs, gut disease or other rare conditions (44–48).

As with other genetic disorders, identification of a mutation has profound implications for family members. The presence of a genetic defect may allow early diagnosis and prompt commencement of SCIG/IVIG treatment of affected family members, when they develop symptoms. We suggest patients with CVID-like disorders are offered SCIG/IVIG on the basis of clinical symptoms and vaccine challenge responses may not be necessary. Such pre-symptomatic individuals, carrying the family mutation, could be made aware of potential risks and complications. This may either prevent catastrophic infections and mitigate ongoing target organ damage leading to bronchiectasis and other disabling complications (24).

Detection of a causative mutation may allow a future reduction in the numbers of PIDs by preimplantation genetic diagnosis (PGD). The specific mutation allows prenatal diagnosis with chorionic villus sampling (CVS) and/or PGD. An individualized approach is required. As we have previously stated, it is not appropriate to consider CVS or PGD for families carrying only mutations predisposing to CVID such as *TNFRSF13B*/TACI (49). Even within mutations causing CVID-like disorders, penetrance and expressivity vary widely. Some variants such as the *TCF3* mutation we have described appear to be fully penetrant, while one member of our family with *NFKB1* haploinsufficiency is phenotypically normal. There will need to be careful counseling of such families.

While current technologies will not prevent disease caused by new mutations, PGD could lead to a substantial decrease in the prevalence of disease within a generation. This will result in a major reduction in the burden of suffering as well as healthcare costs. The NZ government offers free *in vitro* fertilization and PGD for families carrying severe genetic defects. Delay in access to this innovative but under-resourced program in NZ is however a significant barrier.

Identification of the specific mutation may lead to new therapeutic options. Patients with mutations of *CTLA-4* or *LRBA* may be candidates for abatacept. Those with gain of function mutations of GOF-*PIK3CD* or loss of function LOF-*PIK3R1* (APDS 1 and 2) may improve with mTOR inhibitors such as rapamycin or newer agents such as Idelalisib. Patients with a severe CVID-like disorder caused by mutations of *ADA2* may benefit from early bone marrow transplantation.

Discovery of the mutation may in the future lead to gene-based therapies including retroviral gene transfer or gene editing with CRISPR-Cas9. CRISPR-Cas9 has been used to repair *CYBB* gene mutations in X-linked chronic granulomatous disease cells (50). As discussed previously, off-target effects of the CRISPR-Cas9 system may limit its *in vivo* use (51), although there has been progress to mitigate these risks (52). We are unaware of any current *in vivo* trials of retroviral gene therapy or CRISPR-Cas9 gene editing studies in patients with CVID-like disorders. Given the variable penetrance an expressivity, such gene-based therapies should only be considered for severely symptomatic individuals in the future.

The use of NGS has resulted in new discoveries including novel mechanisms of disease (32). We have recently shown the existence of quantitative epistasis in a patient with digenic inheritance leading to a CVID-like disorder (49, 53). Epistasis is the synergistic, non-linear interaction of two or more genetic loci leading either to a much more severe disorder or to a completely different phenotype. We have suggested the synergistic interaction of genes is termed quantitative epistasis, while those leading to a different phenotype are termed qualitative epistasis (49).

The proband had mutations of both *TCF3* and *TNFRSF13B*/ TACI genes, which caused a severe defect in antibody production leading to a CVID-like disorder. These two genetic loci lie in tandem, along the immunoglobulin production and isotype switching pathways. The synergistic interaction of these two mutations caused quantitative epistasis, leading to a severe CVID-like disorder. Our study confirms genes such as *TNFRSF13B/*TACI have disease modifying effects on the severity of CVID-like disorders and supports the separation these two groups of mutations. Such digenic patients can only be identified by NGS (16, 54) and are a strong argument for sequencing both groups of genes, either causing CVID-like disorders (*NFKB1, NFKB2*, etc.) or those modifying the severity of CVID, such as *TNFRSF13B/* TACI (19, 55).

## Diagnostic Uncertainty Caused by Variability of Protein Based Laboratory Tests

As discussed above, the diagnosis of CVID relies on diagnostic criteria. In our long-term prospective NZ hypogammaglobulinemia study (NZHS), we have shown marked fluctuations in IgG levels in patients with hypogammaglobulinemia. Of concern was that 41.6% (20/48) of symptomatic patients were able to normalize their IgG on at least one occasion, when measured over time. Seven of twelve hypogammaglobulinemic patients with bronchiectasis were also able to normalize their IgG on at least one occasion. We have termed this phenomenon transient hypogammaglobulinemia of adulthood (THA) (26).

Some of these patients with hypogammaglobulinemia may have a CVID-like disorder and in time will experience progressive clinical deterioration (23). Identifying a causative genetic defect will establish the diagnosis and assist with monitoring and therapeutic decisions. Patients with profound hypogammaglobulinemia (<3 g/l) and those who are symptomatic with persistent hypogammaglobulinemia should be considered for genetic testing. Patients with asymptomatic THA with subsequent sustained normal IgG levels do not need testing, with the possible exception of those with a family history of an immunological disorder. We have shown that some family members carrying mutations of CVID-like disorders can be asymptomatic with normal IgG levels (23). We acknowledge patients with THA will need to be carefully assessed.

Most patients with CVID have reduced memory B cells and these constitute a diagnostic criterion in Category C of our criteria (56). It is however important for memory B cell subsets to be measured on at least two occasions, as we have shown the numbers can vary on repeat testing (57). We assessed memory B cell subsets on a monthly basis in a cohort of CVID patients being treated with IVIG. Our results showed there was considerable variability leading to changes in diagnostic categories on a monthly basis, particularly for the Freiburg and Paris criteria. The variability was less marked for the EUROclass trial guideline. This again illustrates the variability of protein based assays for CVID assessment and is an argument for genetic testing.

## Unreliability of Vaccine Responses in Current Diagnostic Criteria for CVID

We have discussed the difficulties with the previous ESID/PAGID (1999) criteria for CVID (58). They lacked precision and asymptomatic patients with trivial hypogammaglobulinemia of IgG and IgA, with mildly impaired responses to the diphtheria vaccine could be designated as having CVID and offered life-long SCIG/IVIG.

We have recently reviewed diagnostic criteria for CVID in the NZHS (26). We showed while there was general congruence of diagnostic criteria, there were important differences. In our study, many asymptomatic individuals with mild hypogammaglobulinemia qualified as having definite CVID by ICON (2016) criteria, because of impaired vaccine responses to Pneumovax 23^®^ or the diphtheria vaccine (26). Given their excellent health over a mean follow-up of 106 months (to date), it is unlikely these asymptomatic patients have definite CVID or any other immunological disorder.

In our study, both symptomatic and asymptomatic patients with hypogammaglobulinemia had excellent responses to *H. influenzae* type B (HIB) and tetanus vaccines (59). Vaccine responses were thus non-discriminatory in the NZHS. Similarly, we also recently showed some patients with THI, who subsequently recovered, had impaired vaccine responses, which could potentially lead to misdiagnosis of definite CVID if ICON (2016) criteria are applied (27). IgM and IgA levels in particular, can be difficult to interpret in young children.

Identifying the causative mutation would obviate the need to apply CVID diagnostic criteria, as the patient would then be reclassified as having a CVID-like disorder (60). Thus, the primary aim of genetic sequencing is to remove these patients from the umbrella diagnosis of CVID so they can be more accurately classified as having a specific PID.

## Caveats

While we advocate routine diagnostic WES or WGS for all patients with a CVID phenotype, there are important caveats. We have discussed the technical limitations of NGS including lack of uniform coverage with WES leading to errors (19, 61). These errors are less likely with WGS but currently this technology is more expensive than WES. NGS is not available in all parts of the world. However, several commercial companies are now offering these tests, some using gene panels, while others offer WES with targeted analysis. One company offering WES with targeted analysis releases raw data for an additional fee, which allows future analysis of gene mutations, which have yet to be discovered. With the appropriate consents and ethics approvals, this data can also be converted to parents:child trio analysis for gene-discovery research studies.

It is important to counsel patients before offering these studies as there is a risk of identifying variants of unknown significance (VUS) (19). This can be frustrating for both patient and physician (62). In some cases the pathogenicity of a VUS can be resolved by collateral techniques such as functional studies (63). Another important caveat is the risk of assigning disease causality to ethnic specific variants. What may fit all decision criteria for a mutation causing a rare disease may simply be a common benign variant (polymorphism) in an under-surveyed ethnic group. Current databases comprise predominantly Caucasian individuals, while other ethnicities such as Maori are poorly represented.

There is also the problem of *de novo* pathogenic mutations in databases, where the disease is yet to manifest. These will need careful analysis. *In silico* analysis and the frequency of such alleles may indicate their true significance. If the frequency of homozygous healthy individuals is lower than expected (given the variant allele frequency), it may suggest that the homozygous state is disease causing.

In some cases, WES and WGS may identify potentially important mutations in unrelated genes such as those associated with cancer, cardiovascular disease, severe neurological disorders etc. The American College of Medical Genetics (ACMG) has published guidelines for the analysis and disclosure of these “medically actionable” incidental findings in patients undergoing NGS (64). We have discussed the difficulties with these ACMG guidelines (19). Studies have shown low yield from these guidelines (65, 66).

There is also a possibility of identifying VUS in these genes. A single expert may not be able to resolve the significance of all of these variants in different organ systems. This is likely to cause great anxiety and expense in societies without a socialized health system, if there is no insurance coverage. At the time of consent, we encourage our patients to opt out of disclosing these incidental findings in unrelated organ systems.

We have also discussed other social and financial disadvantages of identifying the mutation, such as genetic discrimination in the domains of insurance or employment (39). The Americans with Disabilities Act 1990 (ADA) and the Genetic Information Non-discrimination Act of 2008 (GINA) protects Americans from such discrimination. Such enabling legislation is however not universally enacted in all jurisdictions. In spite of robust legislation protecting individual rights, NZ does not currently have laws forbidding genetic discrimination.

Securing funding for these tests is a common problem. Clinical services and insurance providers have been slow to recognize the value of such technology and the far-reaching benefits of testing (25). Prevention of a single case could lead to lifetime savings of over $2M, which would fund NGS for a large cohort of CVID patients. If funding is not immediately available from clinical services, in many cases NGS can be undertaken as part of research studies, with the appropriate consents. In many cases, our patients have self-funded these tests.

In spite of these limitations, we believe all patients with a CVID phenotype should now be routinely offered diagnostic NGS sequencing if resources permit. If a causal mutation is not found, such patients can be enrolled in gene discovery research studies with the appropriate consents and ethics approvals (19).

## Author Contributions

All authors listed have made a substantial, direct and intellectual contribution to the work, and approved it for publication.

### Conflict of Interest

The authors declare that the research was conducted in the absence of any commercial or financial relationships that could be construed as a potential conflict of interest.
